# Annotations

**Published:** 1900-01-27

**Authors:** 


### ANNOTATIONS.

### London Water.

In a report issued by Sir William Crookes, F.R.S., and Professor Dewar, F.R.S., it is stated that during the first six months of 1899 the Thames-derived companies' clear-water wells contained on the average 32 bacteria per c.c., while the New River and the River Lea supplies contained respectively 18 and 25. During the second six months the number of bacteria in the waters from the three were respectively 22, 12, and 14; and it is added, "When we consider that a water containing about 100 bacteria per c.c. in the clear-water wells would be regarded by the highest authorities as properly filtered, we see that the London supply must be considered exceptionally good." Some people may see it. We do not. The average number of microbes present has nothing to do with the question any more than the number of microbes met with in the clear-water wells of the companies has, so long as these wells contain the mixed products of several filters. The highest authorities would certainly not regard the fact of a mixed water, or even a water from a very large filter, containing only about 100 microbes per c.c., as any proof that the whole of that water had been well filtered. Everyone admits that on an average the water supplied to Londoners is splendid. But when we find eels getting into water-pipes; and when we recall the gross impurities which have been from time to time met with in the water, it becomes clear enough that a high degree of purity, on the average, is no security against the proof that even the grossest impurities do pass the filters now and again. When a man drinks a glass of the stuff which is now and then served up to him by the water companies it is no comfort or satisfaction to him to be told that, if he struck an average, the water would really have appeared to be of a high degree of purity. Nor can he feel that the company has exonerated itself when it has shown that when the water flowed from the filter it was clean. The pipes belong not to him, but to the company; he is absolutely without control over them; all he can do is to drink what is given him, and if this is dirty no assurances as to its average purity, nor as to its condition as it flows from the filter, are likely to appease his discontent.

### Food Preservatives.

It is satisfactory to be assured that the "official" view is in favour of cleanliness in the preparation, storage, and conveyance of food products, and opposed to the use of preservatives. Dr. Stevenson, analyst to the Home Office, giving evidence before the Departmental Committee which is inquiring into the subject, said that he thought there would be no difficulty in conducting the milk supply of London without preservatives if provision were made for cooling the milk rapidly, employing cleanly methods, and quickening the means of transmission; and he made the important suggestion that experimental work should be undertaken by or on behalf of the committee in order to arrive at correct conclusions on points which were matters of controversy. With this we thoroughly agree. As things stand the committee are face to face with a whole mass of contradictory evidence, and in judging as to its value will no doubt be reduced to the very unsatisfactory expedient of weighing the standing and reliability of the witnesses rather than the value of what they say. The committee will be well advised, then, to adopt the experimental method rather then add to the pile of "evidence," or in other words, of "opinion" which is being offered in such abundance. They must "search out the secrets of nature by way of experiment." As in the days of Harvey, this is the only way. Men who pose as "scientific" are as plentiful as blackberries, and, like blackberries, most of them are unripe. There is no view, however bizarre, which could not be supported and made to wear an air of truth by well-assorted "scientific" evidence—and honest withal. We by no means impugn the honesty even of people who say the world is flat, but if we want to find out whether their evidence is true, we must try for ourselves. So, in regard to the disputed points brought before the committee, it is not evidence and opinions, but facts which are required, and Dr. Stevenson's suggestion that the committee should find out by experiment the points on which there is no doubt a wholesome one.

### Field Hospital Work.

Nothing is more striking in the conduct of the war in South Africa than the display of system and organisation which has been made by the Royal Army Medical Corps in dealing with the wounded at the front. Recognising the importance of wounds not being disturbed more frequently than is absolutely necessary, and of the desirability of not frittering away the time and energies of the staff at the field hospitals on cases too trivial or too severe to be dealt with at the front, the greatest care has been taken that the first dressings shall be applied as carefully as possible, and that every case sent back from the field shall be ticketed with the nature of his injury, besides other necessary details. When we hear that after the battle of Colenso 800 patients passed through the field hospitals in the course of the day, it is easy to see how essential must be the most perfect discipline, and the most complete adherence to regularity and system, if utter confusion is to be avoided. To deal so thoroughly with such numbers was a task of the greatest possible magnitude, and the greatest credit is due to those who were engaged in the work and carried it through successfully. Those who know how great is the strain of operative work will best understand what it means to have been at work in the operating tents, dressing and operating from three o'clock in the morning till far into the evening, without time for either food or rest, and to have been able, notwithstanding the heat, and hurry, and excitement all around, to do their work deliberately, with skill and with efficiency. Among the interesting points which are being demonstrated by the cases that have come under treatment, one must note the comparatively slight injury caused in many cases by bullet wounds of the lung. Case after case is reported in which there seemed to be no doubt that the lung had been traversed, and in which the symptoms were of the slightest, and in regard to some of them it is especially to be noted that the patients, after having been struck, had been able to make considerable exertion. Many of the bullet wounds of the abdomen have also done very well, but in many others it has been impossible to give any material relief. In fact, it seems as if these cases had been either very severe or very slight, those for which operative surgery could do much not having been frequently met with.

